# 3,6-Dichloro­catechol

**DOI:** 10.1107/S1600536808025014

**Published:** 2008-08-13

**Authors:** An-Le Xie, Tong-Jian Ding, Xiao-Ping Cao

**Affiliations:** a State Key Laboratory of Applied Organic Chemistry and College of Chemistry and Chemical Engineering, Lanzhou University, Lanzhou, Gansu 730000, People’s Republic of China

## Abstract

The title compound, C_6_H_4_Cl_2_O_2_, exhibits a two-dimensional supra­molecular hydrogen-bonded network and forms a three-dimensional network supra­molecular structure *via* hydrogen bonds and π–π stacking of benzene rings. The π–π inter­actions are between the benzene rings of centrosymmetrically related mol­ecules, with centroid–centroid distances of 3.7676 (13) and 3.7107 (13) Å.

## Related literature

For related literature, see: Haigler *et al.* (1988 ▶); Kirsh & Stan (1994 ▶); Nishizawa & Satoh (1975*a* ▶,*b* ▶); Sander *et al.* (1991 ▶); Schraa *et al.* (1986 ▶); Spiess *et al.* (1995 ▶); Spain *et al.* (1989 ▶).
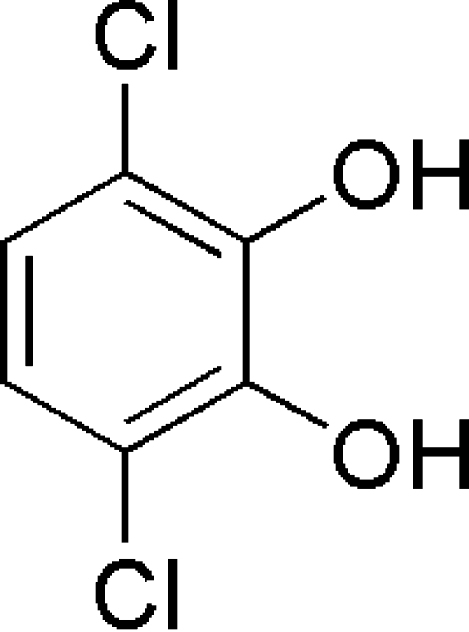


## Experimental

### 

#### Crystal data

C_6_H_4_Cl_2_O_2_
*M* *_r_* = 178.99
Monoclinic, 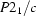

*a* = 7.4411 (7) Å
*b* = 10.1283 (10) Å
*c* = 10.6448 (8) Å
β = 119.903 (5)°
*V* = 695.45 (11) Å^3^
*Z* = 4
Mo *K*α radiation
μ = 0.86 mm^−1^
*T* = 296 K
0.36 × 0.17 × 0.15 mm

#### Data collection

Bruker SMART CCD area-detector diffractometer
Absorption correction: multi-scan (*SADABS*; Bruker, 1997 ▶) *T*_min_ = 0.748, *T*_max_ = 0.882
3531 measured reflections
1243 independent reflections
1117 reflections with *I* > 2σ(*I*)
*R*_int_ = 0.017

#### Refinement

*R*[*F* ^2^ > 2σ(*F* ^2^)] = 0.030
*wR*(*F* ^2^) = 0.118
*S* = 1.01
1243 reflections
93 parameters
H-atom parameters constrained
Δρ_max_ = 0.18 e Å^−3^
Δρ_min_ = −0.34 e Å^−3^

### 

Data collection: *SMART* (Bruker, 1997 ▶); cell refinement: *SMART*; data reduction: *SAINT* (Bruker, 1997 ▶); program(s) used to solve structure: *SHELXS97* (Sheldrick, 2008 ▶); program(s) used to refine structure: *SHELXL97* (Sheldrick, 2008 ▶); molecular graphics: *SHELXTL* (Sheldrick, 2008 ▶); software used to prepare material for publication: *SHELXTL*.

## Supplementary Material

Crystal structure: contains datablocks global, I. DOI: 10.1107/S1600536808025014/bq2078sup1.cif

Structure factors: contains datablocks I. DOI: 10.1107/S1600536808025014/bq2078Isup2.hkl

Additional supplementary materials:  crystallographic information; 3D view; checkCIF report

## Figures and Tables

**Table 1:** Hydrogen-bond geometry (Å, °)

*D*—H⋯*A*	*D*—H	H⋯*A*	*D*⋯*A*	*D*—H⋯*A*
O1—H1⋯O2	0.82	2.19	2.6391 (17)	115
O1—H1⋯Cl1^i^	0.82	2.76	3.3980 (16)	137
O2—H2⋯Cl2	0.82	2.61	3.0597 (13)	116
O2—H2⋯O1^ii^	0.82	2.13	2.8969 (19)	155

Symmetry codes: (i) 
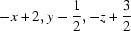
; (ii) 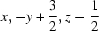.

**Fig. 1.**
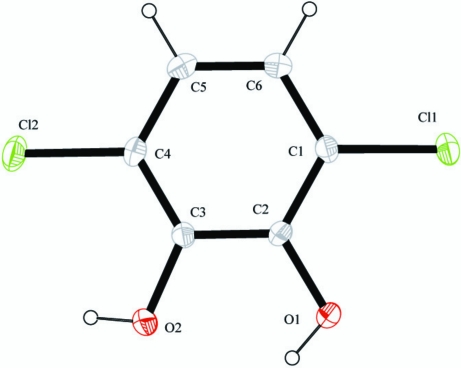
A view of the title compound, showing the atom-numbering scheme. Displacement ellipsoids are drawn at the 50% probability level.

**Fig. 2.**
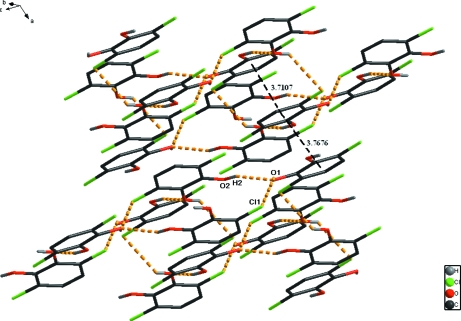
The three-dimensional structure by molecular packing, showing the intermolecular hydrogen bonds as yellow dashed lines [Symmetry codes: (i) -x+2, y-1/2, -z+3/2; (ii) x, -y+3/2, z-1/2], and π-π interactions as black dashed lines.

## supplementary crystallographic information

### Comment

The compound 3,6-dichlorocatechol, (I), was a common metabolite in the microbial aerobic degradation of 1,4-dichlorobenzene. Because 1,4-dichlorobenzene was too stable to be degraded by photochemistry, biodegradation of this compound was an only way that it was eliminated from enviroment. 3,6-Dichlorocatechol has been reported to be an important intermediate in this process (Haigler *et al.*, 1988; Schraa *et al.*, 1986; Spain *et al.*, 1989; Sander *et al.*, 1991; Spiess *et al.*, 1995). So the title compound (I) could be used to optimize the biodegradation process of 1,4-dichlorobenzene (Kirsh *et al.*, 1994). It would be of great important significance in the protection of our surrounding and public health. Herein, we report  the synthesis and structure of this compound, namely 3,6-dichlorocatechol. As shown in Fig.1, there are two hydroxyl groups in the phenyl ring. In the formation of these hydrogen bonds, one acts as donor, the other as acceptor. A two-dimensional supramolecular network was formed by  O—H···Cl and  O—H···O  intermolecular hydrogen bonds (Table 1) [Symmetry codes (i): -x+2, y-1/2, -z+3/2; (ii): x, -y+3/2, z-1/2], and there are also weak π-π interactions between the centrosymmetrically related phenyl rings at (x, y, z) and (-x, -y, -z+1), (-x+1, -y, -z+1) with a centroid-to-centroid distance of 3.7676 (13)Å and 3.7107 (13)Å, respectively (Fig. 2).

### Experimental

3,6,6-Tricholor-2-hydroxycyclohex-2-en-1-one (26 g, 0.12 mol) was treated with Li_2_CO_3_ (13.4 g, 0.18 mol) in DMF to give the title compound (I). (18.4 g) in 86% yield (Nishizawa & Satoh, 1975*a*,*b*). m. p. 108-109°C; ^1^H NMR (CDCl_3_, 300 MHz) δ: 5.79 (s, 2H), 6.86 (d, J = 2.4 Hz, 2H); ^13^ C NMR (CDCl_3_, 75 MHz) δ: 118.7, 120.8, 140.6; MS (ESI) m/*z* (%): 178 (*M*+, 95), 180 (49), 182 (8).

### Refinement

All H atoms were placed in geometrically idealized positions, with C—H = 0.93 Å and O—H = 0.82 Å, and constrained to ride on their respective parent atoms, with *U*_iso_(H) = 1.2*U*_eq_(C) and *U*_iso_(H) = 1.5*U*_eq_(O).

### Crystal data

**Table:**

C_6_H_4_Cl_2_O_2_	*F*_000_ = 360
*M**_r_* = 178.99	*D*_x_ = 1.710 Mg m^−^^3^
Monoclinic, *P*2_1_/*c*	Mo *K*α radiation λ = 0.71073 Å
Hall symbol: -P 2ybc	Cell parameters from 2193 reflections
*a* = 7.4411 (7) Å	θ = 2.9–26.4º
*b* = 10.1283 (10) Å	µ = 0.86 mm^−^^1^
*c* = 10.6448 (8) Å	*T* = 296 K
β = 119.903 (5)º	Block, colorless
*V* = 695.45 (11) Å^3^	0.36 × 0.17 × 0.15 mm
*Z* = 4	

### Data collection

**Table:**

Bruker SMART CCD area-detector diffractometer	1243 independent reflections
Radiation source: fine-focus sealed tube	1117 reflections with *I* > 2σ(*I*)
Monochromator: graphite	*R*_int_ = 0.017
*T* = 296 K	θ_max_ = 25.2º
φ and ω scans	θ_min_ = 3.0º
Absorption correction: multi-scan(SADABS; Bruker, 1997)	*h* = −8→8
*T*_min_ = 0.748, *T*_max_ = 0.882	*k* = −9→12
3531 measured reflections	*l* = −12→12

### Refinement

**Table:**

Refinement on *F*^2^	Secondary atom site location: difference Fourier map
Least-squares matrix: full	Hydrogen site location: inferred from neighbouring sites
*R*[*F*^2^ > 2σ(*F*^2^)] = 0.030	H-atom parameters constrained
*wR*(*F*^2^) = 0.118	*w* = 1/[σ^2^(*F*_o_^2^) + (0.01*P*)^2^] where *P* = (*F*_o_^2^ + 2*F*_c_^2^)/3
*S* = 1.01	(Δ/σ)_max_ = 0.001
1243 reflections	Δρ_max_ = 0.18 e Å^−^^3^
93 parameters	Δρ_min_ = −0.34 e Å^−^^3^
Primary atom site location: structure-invariant direct methods	Extinction correction: none

### Special details

**Table:**

Geometry. All e.s.d.'s (except the e.s.d. in the dihedral angle between two l.s. planes)are estimated using the full covariance matrix. The cell e.s.d.'s are takeninto account individually in the estimation of e.s.d.'s in distances, anglesand torsion angles; correlations between e.s.d.'s in cell parameters are onlyused when they are defined by crystal symmetry. An approximate (isotropic)treatment of cell e.s.d.'s is used for estimating e.s.d.'s involving l.s. planes.
Refinement. Refinement of *F*^2^ against ALL reflections. The weighted *R*-factor *wR* andgoodness of fit *S* are based on *F*^2^, conventional *R*-factors *R* are basedon *F*, with *F* set to zero for negative *F*^2^. The threshold expression of*F*^2^ > σ(*F*^2^) is used only for calculating *R*-factors(gt) *etc*. and isnot relevant to the choice of reflections for refinement. *R*-factors basedon *F*^2^ are statistically about twice as large as those based on *F*, and *R*-factors based on ALL data will be even larger.

### Fractional atomic coordinates and isotropic or equivalent isotropic displacement parameters (Å^2^)

**Table:**

	*x*	*y*	*z*	*U*_iso_*/*U*_eq_	
Cl1	0.84969 (7)	1.04043 (6)	0.81747 (4)	0.0483 (3)	
Cl2	0.64593 (7)	0.99288 (7)	0.17713 (4)	0.0529 (3)	
O1	0.8451 (2)	0.79625 (12)	0.66807 (12)	0.0487 (4)	
H1	0.8578	0.7331	0.6249	0.073*	
O2	0.7560 (2)	0.76889 (12)	0.39645 (12)	0.0490 (4)	
H2	0.7570	0.7708	0.3198	0.074*	
C1	0.7904 (3)	1.02895 (16)	0.63843 (18)	0.0344 (4)	
C2	0.7954 (2)	0.90633 (16)	0.58289 (15)	0.0335 (4)	
C3	0.7515 (2)	0.89485 (16)	0.44037 (17)	0.0330 (4)	
C4	0.7012 (3)	1.00694 (18)	0.35485 (17)	0.0356 (4)	
C5	0.6955 (3)	1.13066 (18)	0.41095 (17)	0.0433 (4)	
H5	0.6619	1.2056	0.3530	0.052*	
C6	0.7397 (3)	1.14090 (18)	0.55181 (19)	0.0415 (4)	
H6	0.7359	1.2230	0.5896	0.050*	

### Atomic displacement parameters (Å^2^)

**Table:**

	*U*^11^	*U*^22^	*U*^33^	*U*^12^	*U*^13^	*U*^23^
Cl1	0.0647 (4)	0.0507 (4)	0.0356 (4)	−0.00327 (18)	0.0296 (3)	−0.00797 (16)
Cl2	0.0633 (4)	0.0666 (5)	0.0307 (4)	0.0071 (2)	0.0248 (3)	0.00907 (18)
O1	0.0832 (10)	0.0343 (7)	0.0401 (7)	0.0122 (6)	0.0393 (7)	0.0089 (5)
O2	0.0835 (10)	0.0351 (7)	0.0414 (7)	−0.0021 (6)	0.0409 (7)	−0.0039 (5)
C1	0.0382 (9)	0.0370 (9)	0.0307 (8)	−0.0029 (6)	0.0192 (7)	−0.0042 (6)
C2	0.0394 (8)	0.0323 (9)	0.0317 (8)	0.0010 (7)	0.0199 (7)	0.0053 (6)
C3	0.0373 (8)	0.0334 (9)	0.0298 (7)	−0.0025 (6)	0.0177 (6)	−0.0020 (6)
C4	0.0360 (9)	0.0445 (10)	0.0276 (8)	−0.0007 (7)	0.0169 (7)	0.0042 (7)
C5	0.0488 (10)	0.0354 (9)	0.0450 (9)	0.0037 (7)	0.0230 (8)	0.0100 (7)
C6	0.0508 (10)	0.0309 (9)	0.0429 (8)	0.0009 (7)	0.0234 (7)	−0.0003 (7)

### Geometric parameters (Å, °)

**Table:**

O1—H1	0.8200	C3—O2	1.365 (2)
O2—H2	0.8200	C3—C4	1.385 (2)
C1—C2	1.384 (2)	C4—C5	1.398 (2)
C1—C6	1.390 (2)	C4—Cl2	1.7299 (16)
C1—Cl1	1.7326 (17)	C5—C6	1.369 (3)
C2—O1	1.3666 (18)	C5—H5	0.9300
C2—C3	1.388 (2)	C6—H6	0.9300
			
O1—C2—C1	120.31 (13)	C3—C4—C5	120.65 (15)
O1—C2—C3	119.68 (14)	C3—C4—Cl2	119.36 (13)
O2—C3—C4	125.85 (14)	C4—C3—C2	119.27 (15)
O2—C3—C2	114.84 (14)	C4—C5—H5	120.2
C1—C2—C3	120.02 (14)	C5—C4—Cl2	119.98 (13)
C1—C6—H6	119.9	C5—C6—C1	120.16 (16)
C2—O1—H1	109.5	C5—C6—H6	119.9
C2—C1—C6	120.32 (15)	C6—C5—C4	119.59 (15)
C2—C1—Cl1	118.94 (12)	C6—C1—Cl1	120.75 (13)
C3—O2—H2	109.5	C6—C5—H5	120.2
			
Cl1—C1—C2—O1	0.4 (2)	C1—C2—C3—C4	−0.5 (2)
Cl1—C1—C2—C3	−179.06 (12)	C2—C1—C6—C5	−0.3 (3)
Cl1—C1—C6—C5	179.23 (13)	C2—C3—C4—C5	0.3 (2)
Cl2—C4—C5—C6	−179.92 (13)	C2—C3—C4—Cl2	−179.90 (12)
O1—C2—C3—O2	2.1 (2)	C3—C4—C5—C6	−0.1 (3)
O1—C2—C3—C4	179.98 (15)	C4—C5—C6—C1	0.2 (3)
O2—C3—C4—C5	177.97 (16)	C6—C1—C2—O1	−179.97 (15)
O2—C3—C4—Cl2	−2.3 (2)	C6—C1—C2—C3	0.5 (2)
C1—C2—C3—O2	−178.41 (15)		

### Hydrogen-bond geometry (Å, °)

**Table:**

*D*—H···*A*	*D*—H	H···*A*	*D*···*A*	*D*—H···*A*
O1—H1···O2	0.82	2.19	2.6391 (17)	115
O1—H1···Cl1^i^	0.82	2.76	3.3980 (16)	137
O2—H2···Cl2	0.82	2.61	3.0597 (13)	116
O2—H2···O1^ii^	0.82	2.13	2.8969 (19)	155

Symmetry codes: (i) −*x*+2, *y*−1/2, −*z*+3/2; (ii) *x*, −*y*+3/2, *z*−1/2.
